# Supplementary material for: Seismic and gravity constraints on plate flexure and mantle rheology along the whole Hawaiian-Emperor seamount chain
Source: Nat Commun. 2025 Dec 2;16:11013. doi: 10.1038/s41467-025-65442-3 (PMC12695922; doi:10.1038/s41467-025-65442-3)
Supplement: Supplementary file 1 — Supplementary Information [file 41467_2025_65442_MOESM1_ESM.pdf]

Supplementary

Seismic and gravity constraints on plate flexure and mantle rheology along the whole Hawaiian-Emperor seamount chain

A. B. Watts[1, ‡], C. Xu[1,2, ‡], P. Wessel[†], D. J. Shillington[3], B. Boston[4], R. Dunn[5]

[1]Department of Earth Sciences, University of Oxford, Oxford, UK

[2]Key Lab of Submarine Geosciences and Prospecting Techniques, Ministry of Education, and College of Marine Geosciences, Ocean University of China, Qingdao, China

[†]Deceased

[3]School of Earth and Sustainability, Northern Arizona University, Flagstaff, AZ, USA.

[4]Auburn University, Department of Geosciences, Auburn, AL, USA

[5]Department of Earth Sciences, School of Ocean and Earth Science and Technology, University of Hawaii at Manoa, Honolulu, HI, USA.

[‡]Authors contributed equally
Email: tony.watts@earth.ox.ac.uk; chong.xu@earth.ox.ac.uk

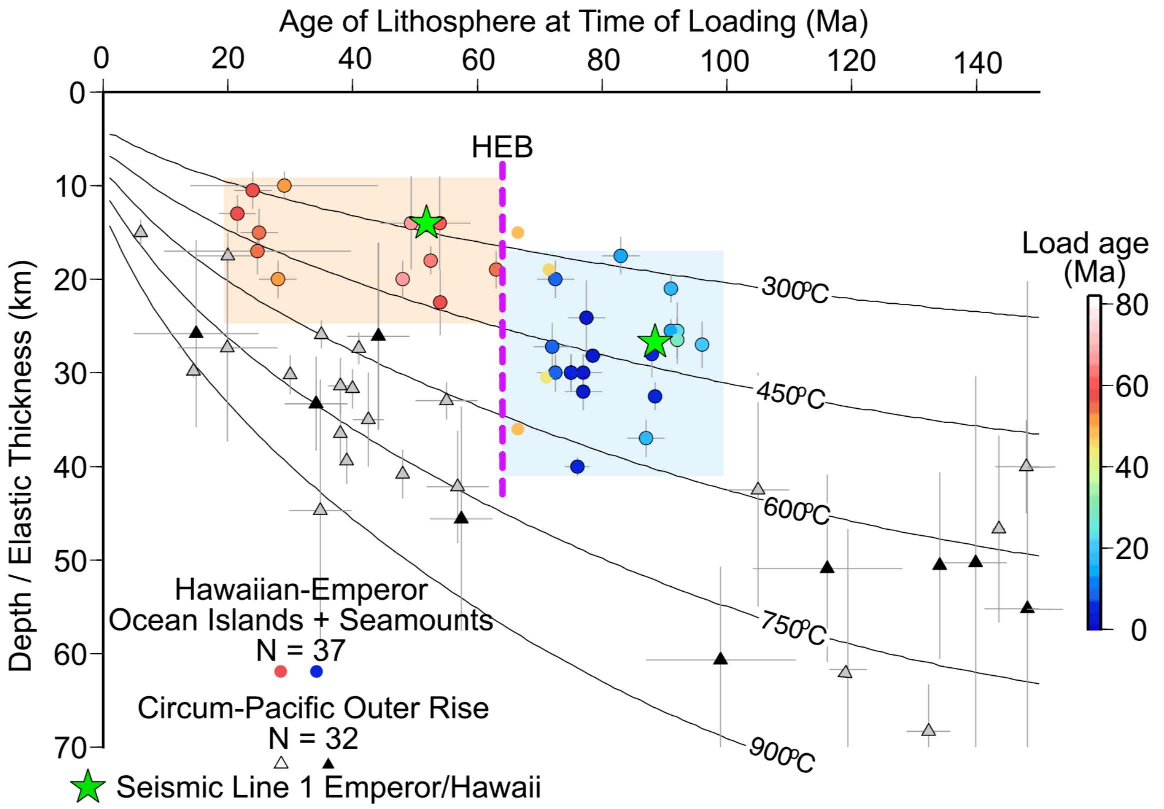

Figure S1. Comparison of previous estimates of T_e along the Hawaiian-Emperor seamount chain (HESC) (circles with colour fill based on age) to previous estimates of T_e at the Circum-Pacific Trench-Outer Rise (CPTOR) (triangles with grey and black fill). The light brown and light blue shading highlight values at the Emperor Seamounts and Hawaiian Ridge respectively. Previous T_e estimates are based on the compilation in Table 6.1 of Watts[52]. Black filled triangles are based on Hunter and Watts[21]. Green filled stars are based on recent seismically constrained estimates at the Emperor Seamounts[30] and Hawaiian Ridge[31]. Thin gray lines show estimated errors in T_e and age of the lithosphere at the time of loading. All estimates show an error in T_e but only some estimates show an error in age. Thin black lines show the depth to the 300°C, 450°C, 600°C, 750°C and 900°C oceanic isotherms based on a cooling plate model[40].

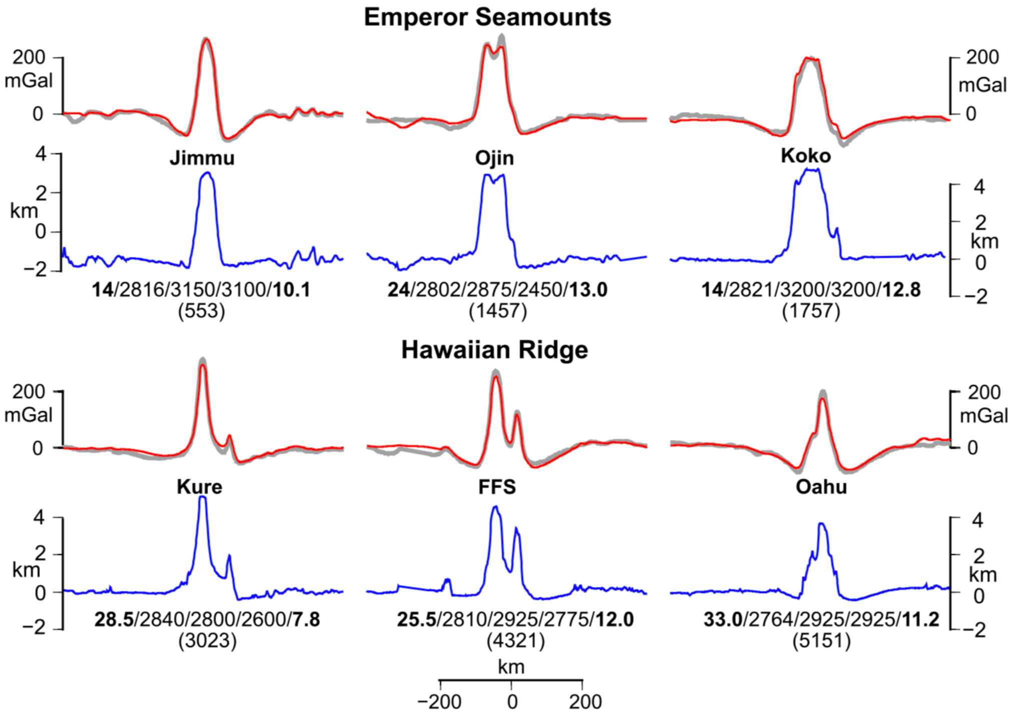

Figure S2. Comparison of selected observed and calculated gravity anomalies based on Model C along cross-sections of the Hawaiian-Emperor seamount chain at 553, 1457, 1757, 3023, 4321 and 5151 km distance along the HESC from Detroit Seamount (Figure 1, inset). FFS = French Frigate Shoals. Grey lines show the satellite-derived free-air gravity anomaly[66]. Blue lines show the bathymetry. Red lines show the calculated gravity anomaly based on Model C (γ = 1.00). Numbers below the bathymetry profiles show the best fit values (from left to right) of T_e (km, bold font), load density (kg m^{-3}), root infill density (kg m^{-3}), moat infill density (kg m^{-3}) and the Root Mean Square (RMS) difference (mGal, bold font) between observed and calculated gravity anomaly.

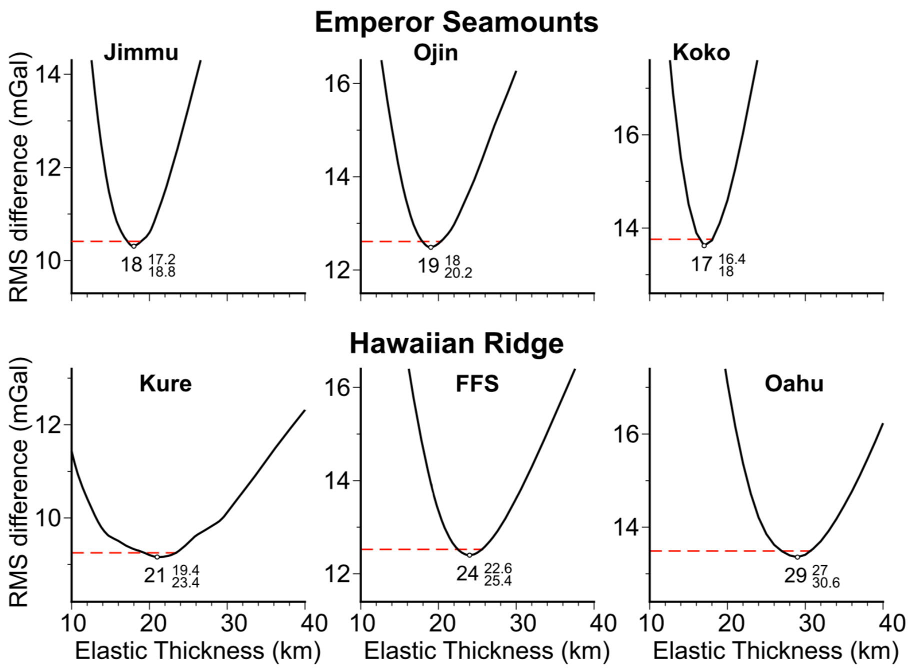

Figure S3. RMS difference between observed and calculated gravity anomalies along cross-sections of the Hawaiian-Emperor seamount chain at 553, 1457, 1757, 3023, 4321 and 5151 km distance along the HESC from Detroit Seamount (Figure 1, inset) as a function of elastic thickness, T_e. The observed gravity anomaly is based on the satellite-derived free-air gravity anomaly[66]. The calculated gravity anomaly is based on the best fit T_e for Model D. The dashed red line shows the lower and higher bounds of T_e which is defined by the points of intersection where the RMS at the minima has increased by 0.10, the tolerance parameter.

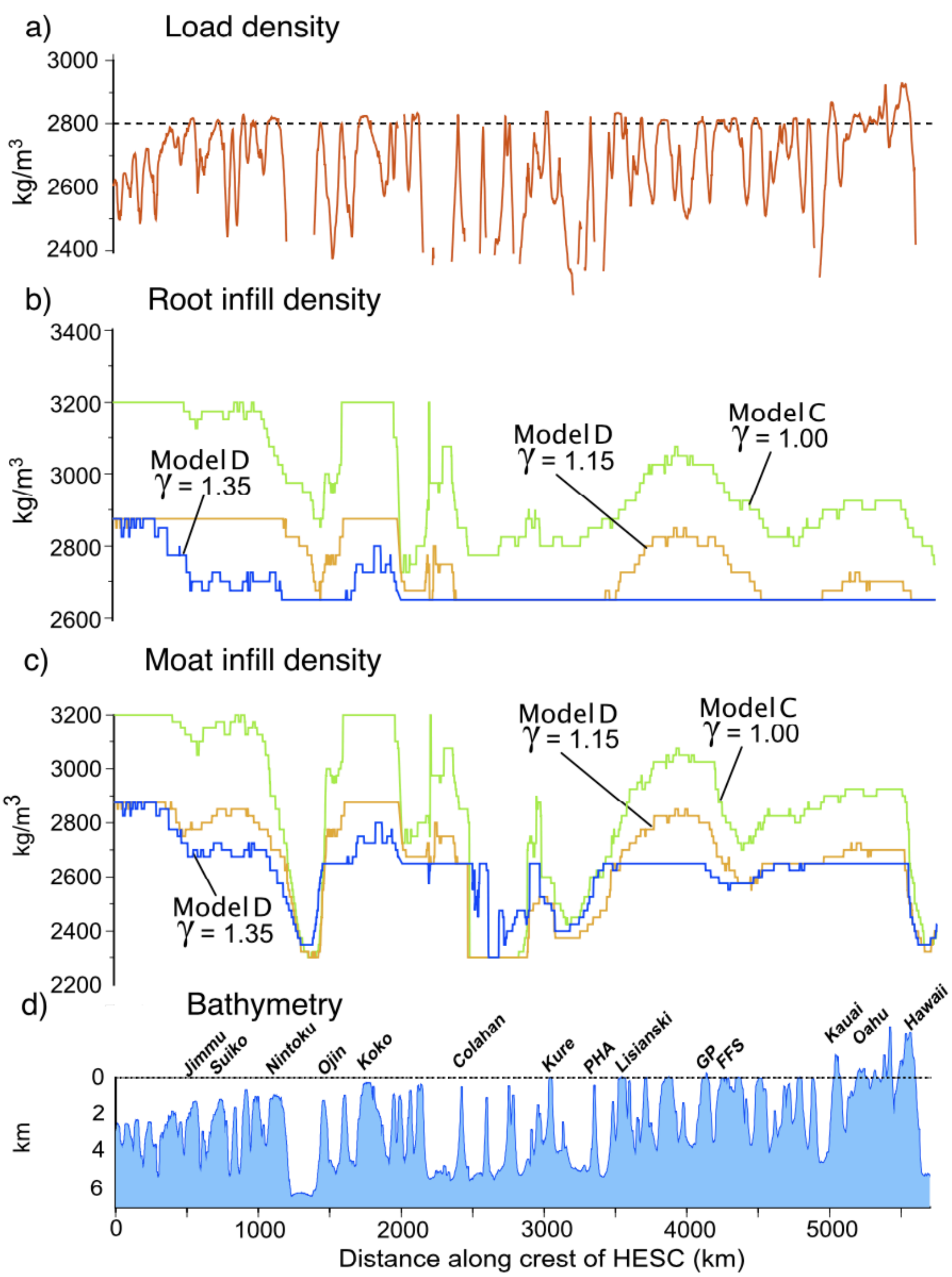

Figure S4. Load and infill densities deduced from gravity and flexure modeling along the Hawaiian-Emperor seamount chain. a) Load density in kg m^{-3} derived from Model C. Note densities reach their peak values over the crest of the seamounts and ocean islands and decrease rapidly over their flanks, as is implied by their *P*-wave velocity structure in Figure 4. b) Root infill density for Models D and C. Note Model D achieves the best fit with a 'load boost' γ = 1.35 while Model C with no 'load boost' (γ = 1.00) achieves it with an increase in the density of the root infill. c) Moat infill density for Models D and C. d) Bathymetry[65]

along the 'trail' that connects summits of individual seamounts and ocean islands that comprise the HESC.

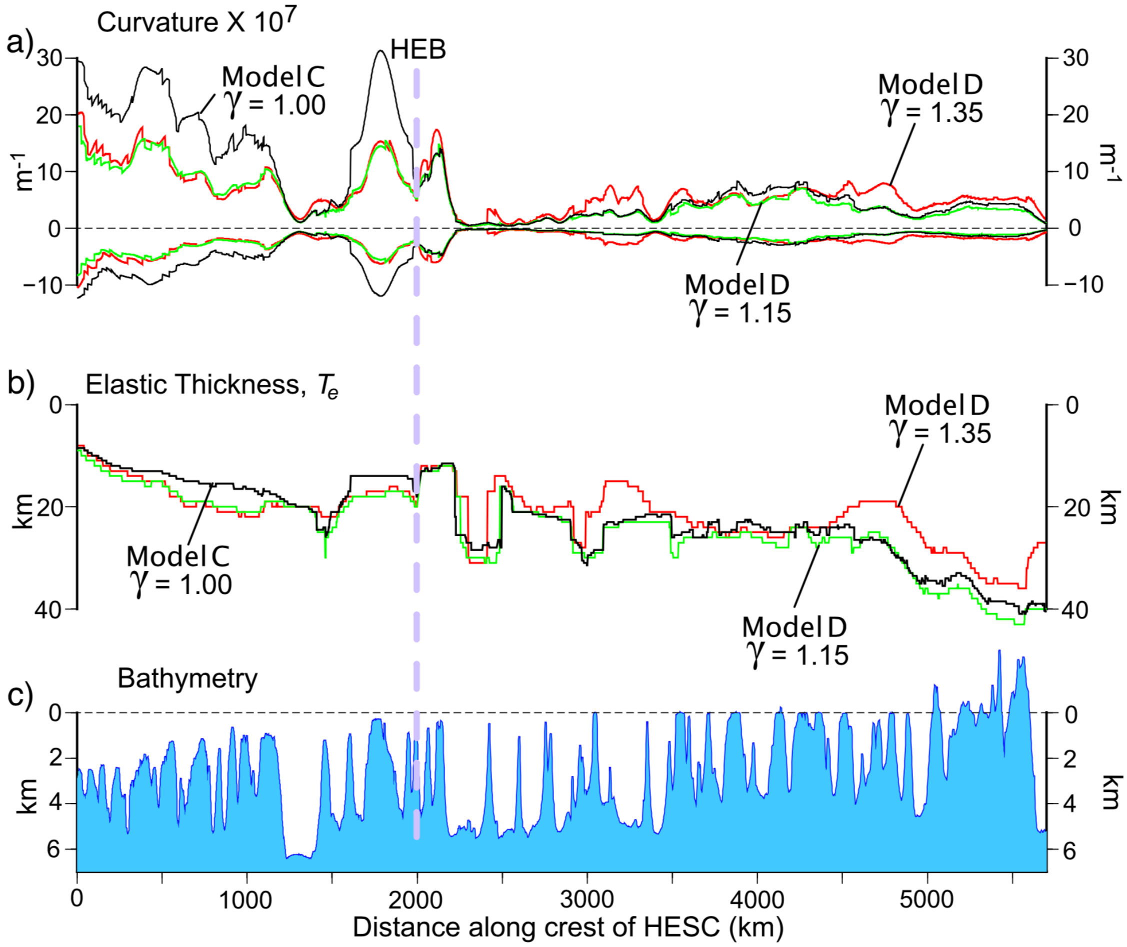

Figure S5. Comparison of the curvature and elastic thickness, T_e, for Models D and C and different values of the 'load boost', γ, along the HESC. a) Maximum positive and maximum negative curvature × 10^7 m^{-1} for Models C (γ = 1.00) and D (γ = 1.15 and γ = 1.35). b) Elastic thickness derived from gravity, bathymetry and flexure and Models D and C. c) Bathymetry along the axis of the HESC derived from the SRTM15 V2.4[65] 15 × 15 arcsec bathymetric grid.

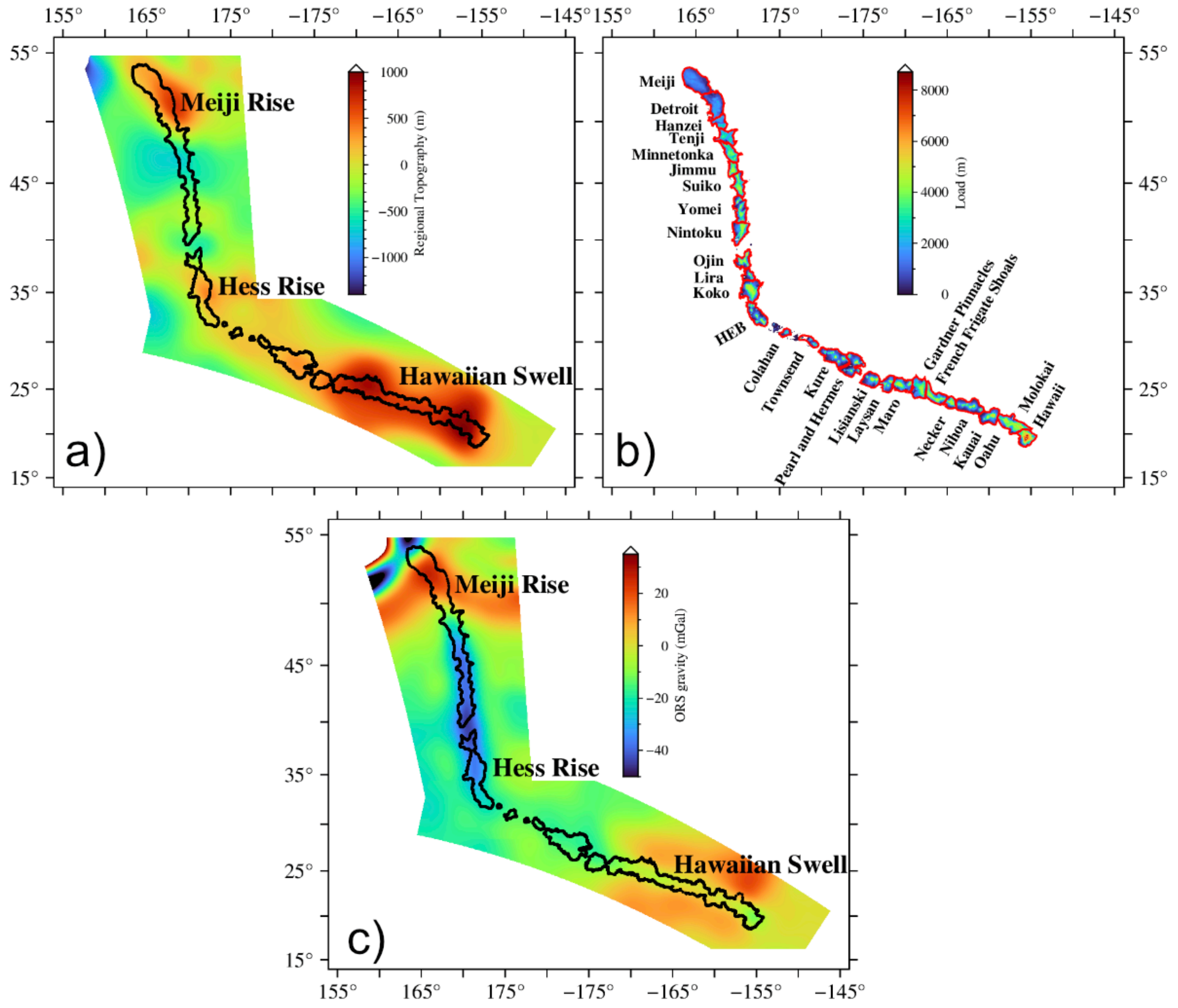

Figure S6. Application of the Optimum Robust Separator (ORS)[37,88] to bathymetry and gravity anomaly data in the vicinity of the Hawaiian-Emperor seamount chain. a) Regional bathymetry obtained by application of an ORS filter of width 346 km for the Emperor Seamounts and 450 km for the Hawaiian Ridge to the bathymetry data based on SRTM15+V2.4[65]. b) The 'driving' load for 27 islands and seamount loads (including atolls and guyots) obtained by subtracting the regional bathymetry from the observed bathymetry along the Hawaiian-Emperor seamount chain. c) Regional gravity anomaly obtained by application of an ORS filter of width 346 km for the Emperor Seamounts and 450 km for the Hawaiian Ridge to the gravity anomaly data based on V29.1[66].

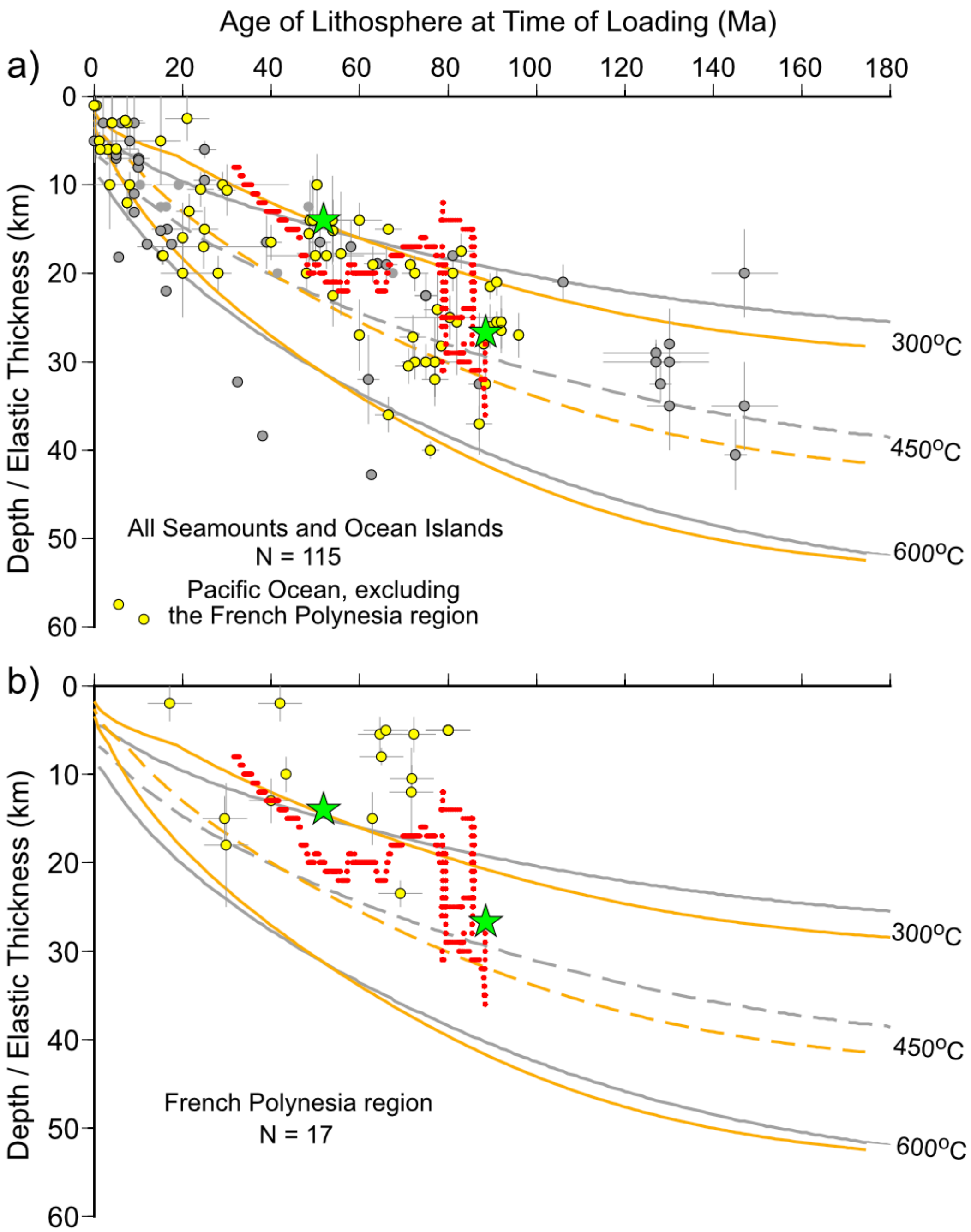

Figure S7. Plot of Elastic thickness, T_e, against age of the lithosphere at the time of loading showing a comparison of Model D results (red filled circles) with previous estimates from seamounts and oceanic islands[52]. Green filled stars show estimates based on seismic reflection and refraction data at the Emperor Seamounts[30] and Hawaiian Ridge[31]. Solid lines show the depth to the 300°C, 450°C and 600°C oceanic isotherms based on the cooling plate models of Parsons and Sclater[40] (Grey lines) and McKenzie et al.[89] (Orange lines). a) Seamounts and oceanic islands from the Atlantic, Indian and Pacific Oceans. Yellow filled circles highlight estimates from the Pacific Ocean, excluding estimates from the French

Polynesia region or seamounts and ocean islands that backtrack to the region. b) Seamounts and oceanic islands from the French Polynesia region of the Pacific Ocean. Note that T_e results of Model D overlap those of previous estimates in the Pacific Ocean, except those from the French Polynesia region, which is associated with the South Pacific Isotopic and Thermal Anomaly (SOPITA)[90].

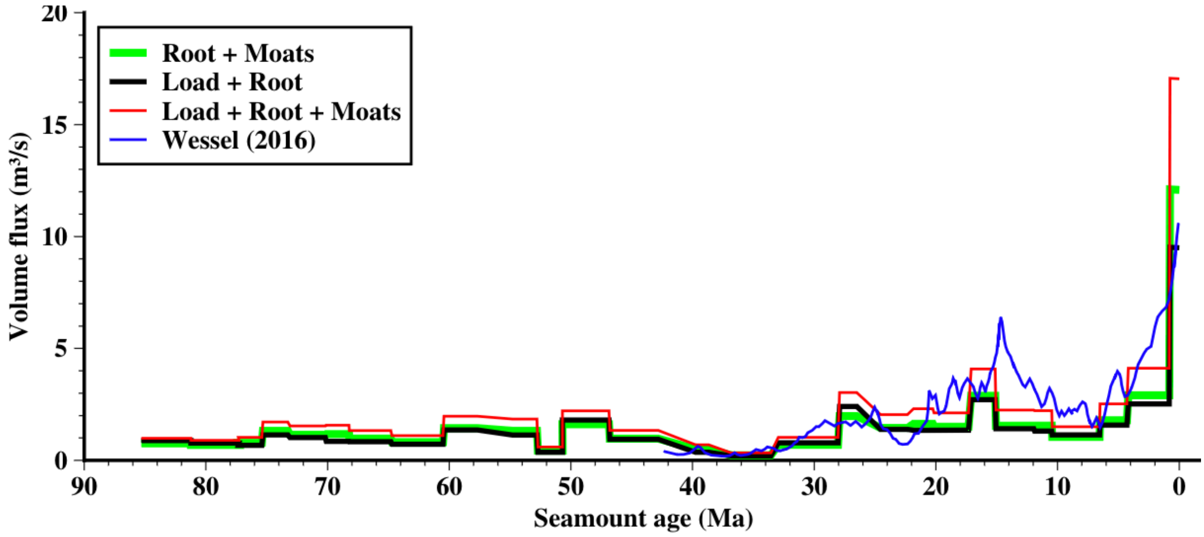

Figure S8. Volume flux as a function of seamount and ocean island age along the HESC. Thick black line shows the flux based on the sum of the 'driving' load and the root infill load. We believe this to be a minimum estimate of the volume flux associated with magmatic (extrusive + intrusive) material as it does not include any magmatic material that might have filled the flexural moats. Red line shows the maximum possible volume flux associated with magmatic material. Blue line shows the volume flux estimates of Wessel[37] for comparison.

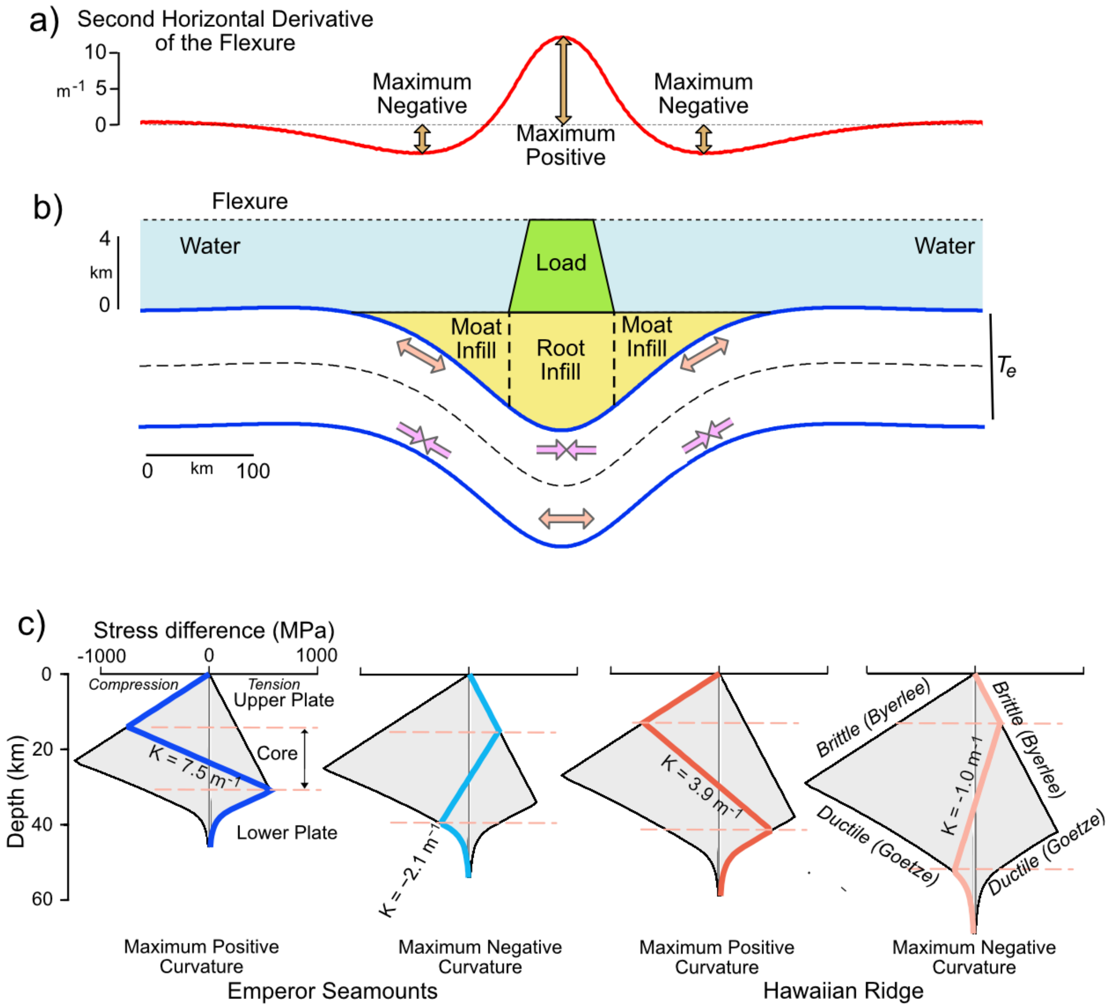

Figure S9. Simple models for the flexure, curvature of flexure, and Yield Strength Envelope (YSE) for surface loading of a thin 2D elastic plate that overlies an inviscid substratum. a) The maximum and minimum curvature of flexure for a Cartesian coordinate system. Curvatures are $\times 10^{7}$ m^{-1}. b) The 'driving' load (green shading), the moat and root infill loads (yellow shading) and the flexure (solid blue lines). Arrows schematically illustrate the stress state in the uppermost and lowermost part of the flexed plate. The thin dashed line shows the neutral surface, which is assumed fixed during flexure. c) The YSE based on a brittle[16] and ductile[17,18] rheology for a maximum positive curvature and a maximum negative curvature and for an age of the plate at the time of loading corresponding to the Emperor Seamounts (Blue and light blue lines) and the Hawaiian Ridge (Red and light red lines) of 50 Ma and 90 Ma respectively.

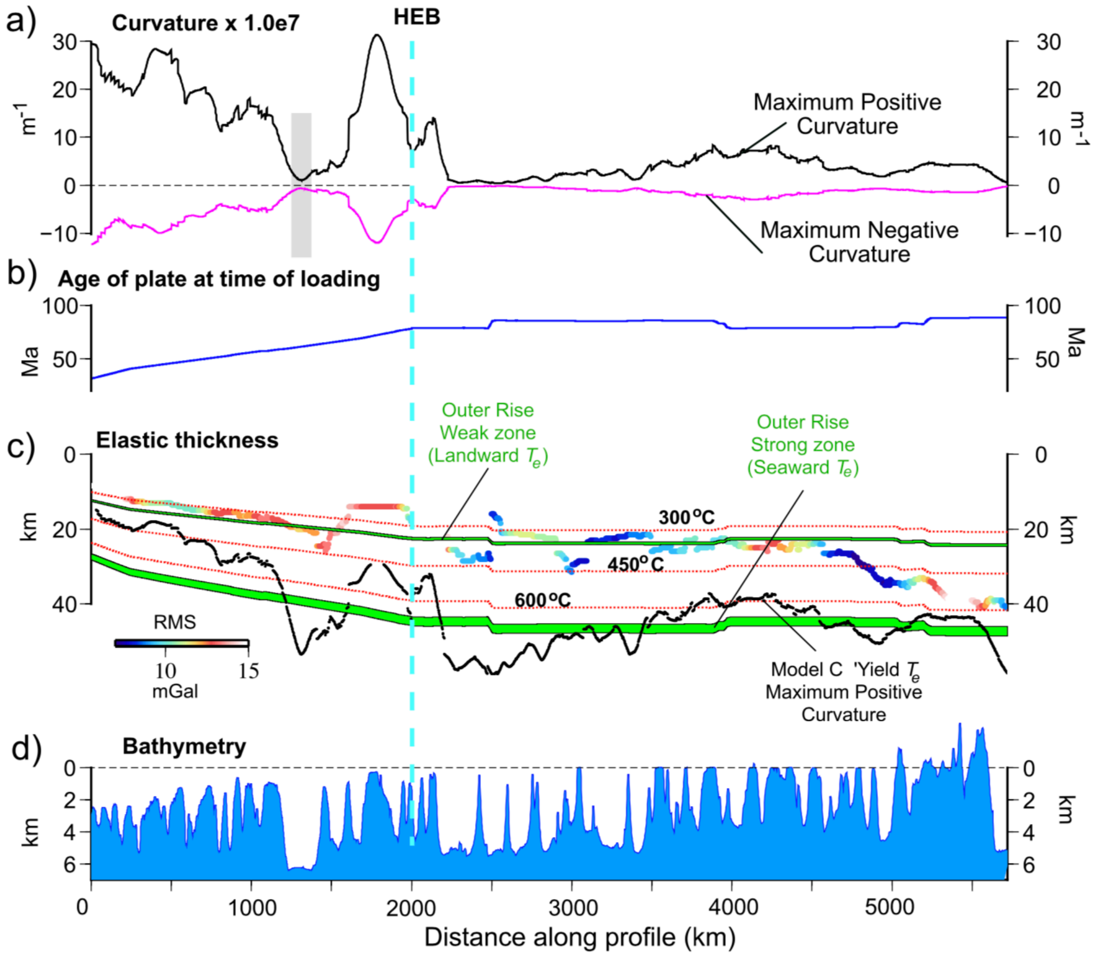

Figure S10. The curvature of flexure and T_e along the HESC based on Model C ($\gamma = 1.00$). a) The curvatures (maximum positive, maximum negative) of flexure due to individual seamount and ocean island loads. b) age of the Pacific oceanic plate at the time of loading. c) Comparison of the observed T_e (coloured according to the RMS difference between the observed and calculated gravity) to the 'yield T_e' (black solid line) derived from a Yield Strength Envelope (YSE) based on brittle[16] and ductile flow laws[17,18] and the observed maximum positive (black line). Thin red dotted lines show the depth to the 300, 450 and 600°C oceanic isotherms based on a cooling plate model[89]. Green filled lines show the depth to the controlling isotherms that best fit T_e in the weak zone (upper curve) and the strong zone (lower curve) at the Circum-Pacific Trench- Outer Rise (CPTOR)[21].

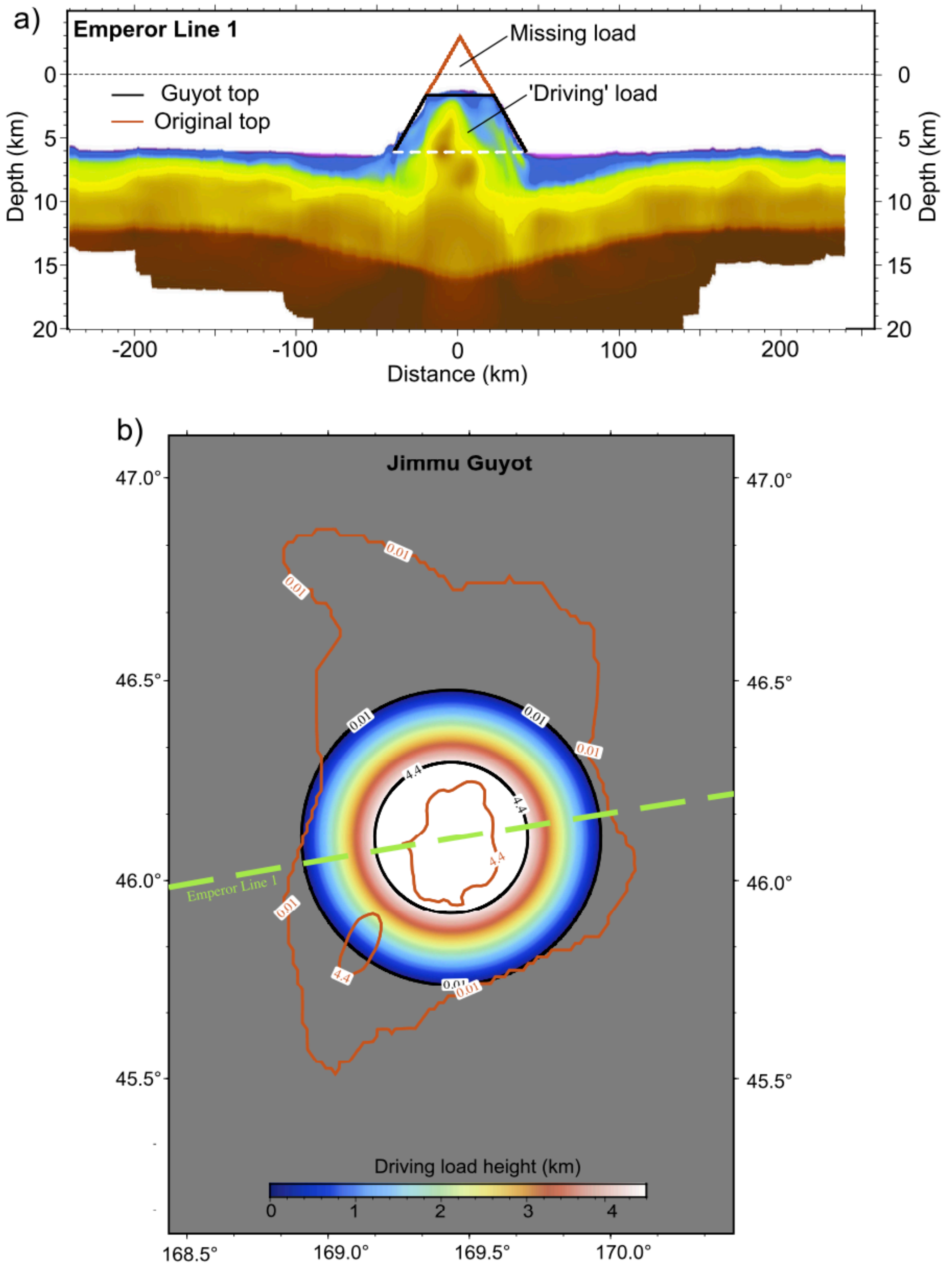

Figure S11. Simple model for the growth of submarine volcano and the load that might be missing due to summit collapse and guyot formation. a) Seismic refraction Line 1 in the Emperor Seamounts (Figure 1) showing the assumed load that drives the flexure. The base of the load was derived from the ORS and is shown as a white dashed line. The top of the load was derived from the top of the guyot and is shown as a black solid line. The red solid line shows the projected height of the seamount derived from the guyot slopes. The region between the black solid line and the red solid line is an estimate of the amount of material that was present at the time of volcano loading and is now missing. b) Planform of the

modelled guyot and its relationship to the actual 'driving' load derived from the bathymetry. Red solid lines show the 0.01 and 4.40 km contours of the actual 'driving' load. Note that by assuming a conical-shape seamount we are only approximating the true shape of the guyot and the missing load.

Volume of missing load in Figure S11 = 2115.360 km^3
Mass of missing load = 2115.360 × 10^9 × ρ_s = 5.923 × 10^{15} kg, assuming ρ_s = 2800 kg m^{-3}
Volume of guyot in Figure S11 = 13727.700 km^3
Mass of guyot = 13727.700 × 10^9 × ($\rho_s - \rho_w$) = 24.298 × 10^{15} kg, assuming ρ_w = 1030 kg m^{-3}
Total mass of seamount/ocean island = 30.221 × 10^{15} kg
∴ Load factor required to boost mass of guyot, γ, = 1.24

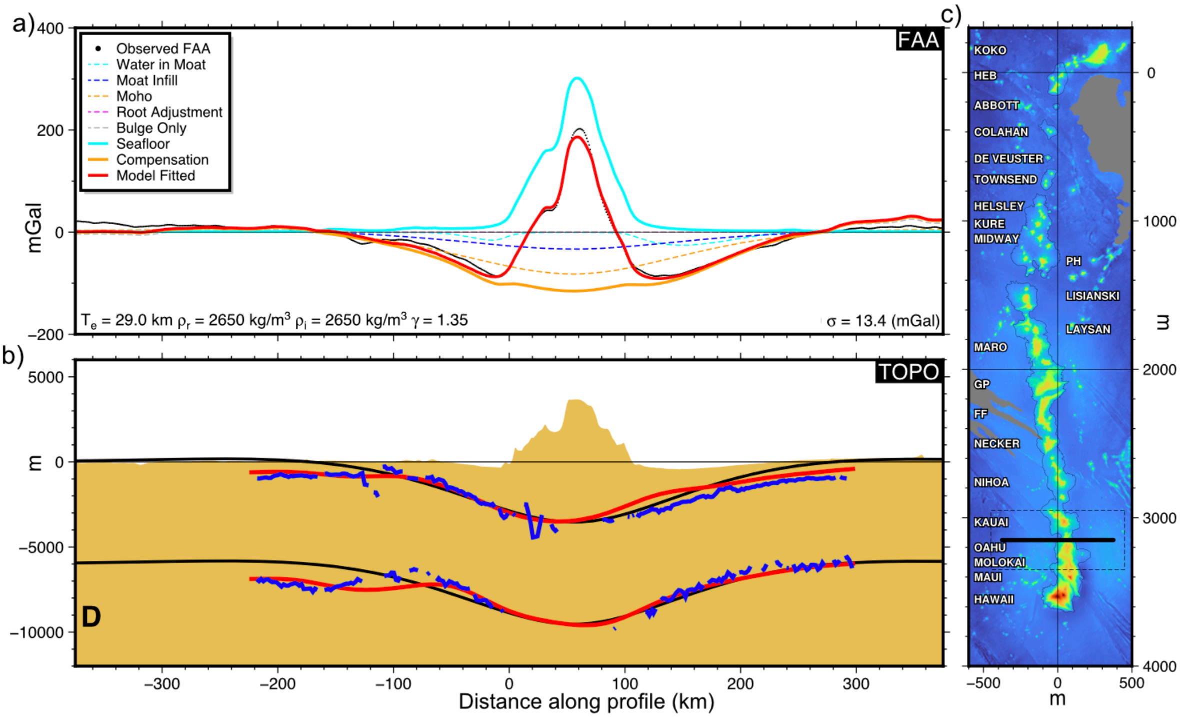

Figure S12. Example of the 7 individual gravity effects associated with the simple model of flexure from Supplementary Movie 2. a) Comparison of observed free-air gravity (dotted black curve) and the best fit calculated gravity anomaly (red curve). Individual curves show the gravity effect of the seafloor, the moat infill, a correction for an unfilled moat infill, the flexural bulge, the flexural depression, and the total effect of the compensation. In this case, the root infill and the moat infill are of the same density and so there is no correction for the root. Numbers show the best fit T_e, load and infill densities, and RMS between observed and calculated gravity anomaly. b) Comparison of the observed surfaces of flexure based on depth converted multichannel seismic reflection (blue dotted curve) and refraction (red curve)

data along Line 2 Hawaiian Ridge[32,33] to calculations based on the simple model of flexure that best fits the gravity anomaly.

Table S1

Summary of best fit load density, infill density and T_e derived from gravity and flexure modeling along the nearest profile to the seismic transects Line 1 of the Emperor Seamounts and Lines 1 and 2 of the Hawaiian Ridge (Figure 1) at Jimmu/Suiko guyot, Oahu/Kauai and Maui/Hawaii.

Seamount/Ocean Island	Average load density (kg m^{-3}) Model C	Average root infill density (kg m^{-3}) Model D	Average moat infill density (kg m^{-3}) Model D	T_e (km) Model D
**Jimmu Guyot**	2817	2675	2675	18
**Oahu/Kauai**	2767	2650	2650	29
**Maui/Hawaii**	2771	2650	2650	35

Table S2

Summary of parameters determined by Goetze and Evans[17,18] for Olivine and used to construct the Yield Strength Envelopes.

Brittle

Coefficient of friction, μ_f = 0.6
Slope of Byerlee failure curve for compression = -54 MPa km^{-1}
Slope of Byerlee failure curve for tension = 18 MPa km^{-1}
Fluid-pore pressure, p_o = 0

Ductile

$$\sigma = (\dot{\varepsilon} * A_p * e^{-Q_p/RT})^n$$

Low stress: Power law
σ < 200 MPa

Stress difference, $\sigma = \sigma$(depth in the lithosphere) MPa
Strain rate, $\dot{\varepsilon} = 10^{-14}$ s^{-1}
Power law exponent, n = 3.5
Power law pre-exponential stress function, $A_p = 7.0 \times 10^{-14}$ Pa^{-n} s^{-1}
Power law activation energy, Q_p = 510 kJ mol^{-1}

Gas constant, $R = 8.3144$ J mol^{-1} K^{-1}

Temperature, $T = T$ (age of the lithosphere at time of loading) Kelvin

High stress: Dorn law

$\sigma > 200$ MPa

$$\sigma = A_d * \left[1 - \left[ln(B_d/\dot{\varepsilon})RT/Q_d\right]^{1/2}\right]$$

Stress difference, $\sigma = \sigma$(depth in the lithosphere) MPa

Strain rate, $\dot{\varepsilon} = 10^{-14}$ s^{-1}

$A_d = 8.5\times 10^9$ Pa

$B_d = 5.7 \times 10^{11}$ s^{-1}

Dorn law activation energy, $Q_d = 535$ kJ mol^{-1}

Gas constant, R = 8.3144 J mol^{-1} K^{-1}

Temperature, $T = T$ (age of the lithosphere at time of loading) Kelvin

Thermal structure

Cooling plate model

Temperature at base of the thermal lithosphere, $T_m = 1333^{\circ}$C

Thermal thickness of the lithosphere, $a = 125$ km

Table S3

Strain rates estimated from the change in thickness as the lithosphere thins from its short-term seismic thickness to its long-term elastic thickness during loading and the time it takes for a seamount or ocean island to form. Note that $\dot{\varepsilon} = 10^{-14}$ s^{-1} implies the time it takes for typical a seamount or ocean island in the HESC to form is a few Myr (~2.45-2.70 Myr) which seems reasonable given the spread of ages from surface samples, although we lack ages from the internal dense cores of seamounts and ocean islands.

	**Depth to 1250°C oceanic isotherm* (km)**	**Average elastic thickness, T_e (km)**	**Average time to form a seamount/ocean island (Myr)**	**Strain rate, $\dot{\varepsilon}$ (s^{-1})**
**Emperor Seamounts**	95	14	0.270	10^{-13}
			2.700	10^{-14}
			27.000	10^{-15}
			270.000	10^{-16}
**Hawaiian**	111.0	24	0.245	10^{-13}

**Ridge**	2.450	10^{-14}
	24.500	10^{-15}
	245.000	10^{-16}

* The depth to 1250°C oceanic isotherm has been interpreted by Audhkhasi and Singh[91] as representative of the seismic Lithosphere/Asthenosphere Boundary (LAB).

References (not cited in main text)

88. Wessel P. An empirical method for optimal robust regional residual separation of geophysical data. *Math Geology*, **30:** 391-408, https://doi.org/10.1023/A:1021744224009 (1998)

89. McKenzie D, Jackson J, Priestley K. Thermal structure of oceanic and continental lithosphere. *Earth Planet Sci Letters*, **233:** 337-349, https://doi.org/10.1016/j.epsl.2005.02.005 (2005)

90. Smith WHF, Staudigel H, Watts AB, Pringle MS. The Magellan Seamounts: Early Cretaceous record of the South Pacific Isotopic and Thermal Anomaly. *J Geophys Res*, **94:** 10,501-510,523, https://doi.org/10.1029/JB094iB08p10501 (1989)

91. Audhkhasi P, Singh SC. Discovery of distinct lithosphere-asthenosphere boundary and the Gutenberg discontinuity in the Atlantic Ocean. *Science Advances*, https://doi.org/10.1126/sciadv.abn5404 (2022)
