# Supplementary material for: Seismic and gravity constraints on plate flexure and mantle rheology along the whole Hawaiian-Emperor seamount chain
Source: Nat Commun. 2025 Dec 2;16:11013. doi: 10.1038/s41467-025-65442-3 (PMC12695922; doi:10.1038/s41467-025-65442-3)
Supplement: Supplementary file 2 — Description of Additional Supplementary File [file 41467_2025_65442_MOESM2_ESM.pdf]

Description of Additional Supplementary Files

File Name: Supplementary Video 1
Description: This video shows the best fits between the observed and calculated free-air gravity anomaly on all 1000 west-east sections of the Emperor Seamount chain beginning at Detroit Seamount in the north and ending at the HEB in the south, together with the gravity effects of the 7 density interfaces that contribute to the calculated anomaly.

File Name: Supplementary Video 2

Description: This video shows the best fits between the observed and calculated free-air gravity anomaly on all 2000 south-north sections of the Hawaiian Ridge beginning at the HawaiianEmperor Bend in the north and ending at the Big Island of Hawaii in the south, together with the gravity effects of the 7 density interfaces that contribute to the calculated anomaly.
